# Cryptogenic Multifocal Ulcerous Stenosing Enteritis: A Review of the Literature

**DOI:** 10.1155/2013/918031

**Published:** 2013-11-24

**Authors:** Darina Kohoutová, Jolana Bártová, Ilja Tachecí, Stanislav Rejchrt, Rudolf Repák, Marcela Kopáčová, Jan Bureš

**Affiliations:** 2nd Department of Internal Medicine-Gastroenterology, Charles University in Praha, Faculty of Medicine at Hradec Králové, University Teaching Hospital, 500 05 Hradec Králové, Czech Republic

## Abstract

Cryptogenic multifocal ulcerous stenosing enteritis (CMUSE) is an extremely rare illness characterised by chronic or relapsing subileus status resulting from multiple small intestinal fibrous strictures and multiple shallow ulcers of the small bowel. The etiology is unknown and pathogenesis is not fully understood. Therapy with systemic glucocorticosteroids is the treatment of choice. However, most patients develop corticosteroid dependence. Deep enteroscopy enables precise diagnostic work, possible endoscopic treatment of stenoses; may obviate the need for surgery and prevent excessive small bowel resections.

## 1. Introduction

Cryptogenic multifocal ulcerous stenosing enteritis (CMUSE) is a rare illness characterised by chronic or relapsing subileus or ileus status resulting from multiple small intestinal fibrous strictures, multiple shallow ulcers of the small bowel, and favourable therapeutic effect of systemic glucocorticosteroids [1–3]. 

## 2. History 

The first descriptions of this rare condition probably came from the late 50s and early 60s [4–9], followed by further case reports or small series [10–17]. Matsumoto et al. [2] informed that Okabe and Sakimura reported first cases of CMUSE in Japan in 1968. Japanese gastroenterologists call this illness chronic nonspecific multiple ulcers (CNSU) of the small intestine [2].

However, all these early reports are difficult to evaluate nowadays, as diagnostic potential was limited at that time. Nowadays, owing to deep enteroscopy, it is possible to investigate the entire small intestine [18–21], take numerous biopsy specimens, and utilise other advanced diagnostic methods like flow cytometry and/or immunohistochemistry. That is why several distinct entities can be newly recognised [3]. 

## 3. Epidemiology

CMUSE is an extremely rare illness. Only about sixty cases of CMUSE have been published so far [1–17, 22–24], but this entity has probably been considerably underdiagnosed or misdiagnosed, mostly with Crohn's disease and nonsteroidal anti-inflammatory drugs- (NSAIDs)-induced enteropathy [3]. Perlemuter et al. [1] reported 12 cases of CMUSE hospitalised in France between 1965 and 1993. This was a retrospective analysis of medical records based on queries sent to 220 French gastroenterology departments. Matsumoto et al. [2] published 15 cases from Japan (1964–2006) and provided thorough review of the literature. Our group reported three cases of CMUSE (diagnosed between 1994 and 2009 in a single tertiary centre) [3]. Chang et al. [18] collected 2 cases of CMUSE diagnosed by means of double balloon enteroscopy in 48 patients (a series from 6 university hospitals in Korea). 

## 4. Etiology and Pathogenesis 

The etiology of CMUSE has not been clarified yet and pathogenesis is still poorly understood [3]. Some authors even doubt the real existence of this entity [18].

Immunological pathogenesis of CMUSE is supported by the favourable therapeutical effect of systemic glucocorticosteroids [3]. Most of these patients become corticodependent [1–3, 24, 25]. The key to the solution of etiology and understanding pathogenesis might be overstimulated production of fibrous tissue [3]. This is a principal sign of CMUSE, responsible for chronic or relapsing subileus episodes. Fibroblast proliferation can be augmented by proinflammatory cytokines (IL-6, IL-8, and TNF-alpha), fibroblast growth factors (FGS II), granulocyte/macrophage colony-stimulating factor (GM-CSF), transforming growth factor beta (TGF-beta), platelet-derived growth factor (PDGF) [26], and endotoxin (lipopolysaccharide) [27]. Other growth factors, such as the connective tissue growth factor (CTGF), which is secreted by fibroblasts and endothelial cells, also promote formation of fibrous tissue [26]. Collagen is degraded by a family of matrix metalloproteinases that include the collagenases. Matrix cells, neutrophils, and macrophages all secrete these proteinases. In healthy people, the degradation of collagen can be very rapid and begins immediately after collagen is produced [28]. We have hypothesized, that multifocal small intestinal disturbance of collagen degradation in CMUSE might play a crucial role in pathogenesis [3]. In CMUSE, fibrous tissue formation can be associated with low or even absent systemic inflammatory response [1].

Perlemuter et al. [1, 30] proposed that CMUSE could be termed as a type of “atypical vasculitis.” However, any type of vasculitis of any cause, if found, should be assigned simply as “vasculitis” not CMUSE [3]. Small intestinal involvement can be found in Churg-Strauss syndrome [31–34], systemic lupus erythematosus [35], Buerger's disease [36], Wegener's granulomatosis [37], Weber-Christian disease [38], and systemic sclerosis [39]. Vasculitis could be associated with multiple small intestinal ulcers and their complications (including perforation), but quite rarely with multifocal stenoses of the small bowel. Furthermore, vasculitis mostly represents a systemic involvement (kidneys, skin, joints, lungs, etc.). None of our three patients revealed any extraintestinal involvement, and small intestinal histology found no signs of vasculitis [3]. Matsumoto et al. [2] did not reveal any arteritis in his series as well. Perlemuter et al. [30] suggested the association of CMUSE with C2 complement deficiency; however, this was not confirmed by other authors [24]. Fraile et al. [40] reported an association of CMUSE with X-linked recessive reticulate pigmentary disorder. CMUSE was also reported in association with homozygous deletion mutations in cytosolic phospholipase A2-*α* [41]. Hussey et al. [42] published a case of CMUSE as a manifestation of enterocolic venopathy. 

## 5. Clinical Features 

Chronic or relapsing subileus episodes resulting from multiple small intestinal strictures are a leading clinical symptom [1–3]. Chronic iron-deficiency anaemia due to small intestinal occult blood loss is frequently found [2, 43]. Patients may present with fatigue, edema, and growth retardation, and they usually have repeated episodes of treatment for anaemia. However, these subjects rarely manifest with diarrhoea, malabsorption, hematochezia or fever. Anaemia may precede the diagnosis of CMUSE for several years [2]. 

Patients with CMUSE were often referred for surgery because of symptomatic small intestinal strictures, and several resections of the small bowel had to be performed [2]. 

Clinicopathological features of CMUSE have been summarised as (1) unexplained small intestinal strictures found in adolescent and middle-aged subjects, (2) superficial ulceration of the mucosa and submucosa, (3) chronic or relapsing clinical course (even after surgery), (4) no biological signs of systemic inflammatory reaction, and (5) beneficial effect of systemic glucocorticosteroids [1, 2]. 

Perlemuter et al. [1] reported 12 cases of CMUSE hospitalised between 1965 and 1993. Despite surgery, symptoms reoccurred in seven of ten patients, and recurrence of strictures was reported in four. Inflammatory infiltrate was made of neutrophils and eosinophils. Glucocorticosteroid therapy was effective, but caused steroid dependence [1]. Chang et al. [18] collected 2 cases of CMUSE diagnosed by means of double balloon enteroscopy in 48 patients (a series from 6 university hospitals in Korea). Both individuals suffered from chronic recurrent abdominal pain. One patient was presented with recurrent melaena (for 41 months), the other one was referred for surgery because of retention of a capsule endoscope in the stenotic site. Mesenteric arteriography did not demonstrate any evidence of arteritis [18].

**Figure 1:**
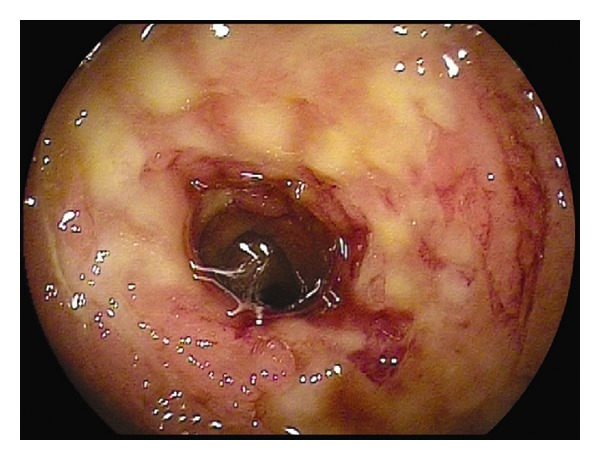
Double balloon enteroscopy. Severe inflammatory small intestinal involvement in front of tight stenosis of the jejunum.

**Figure 2:**
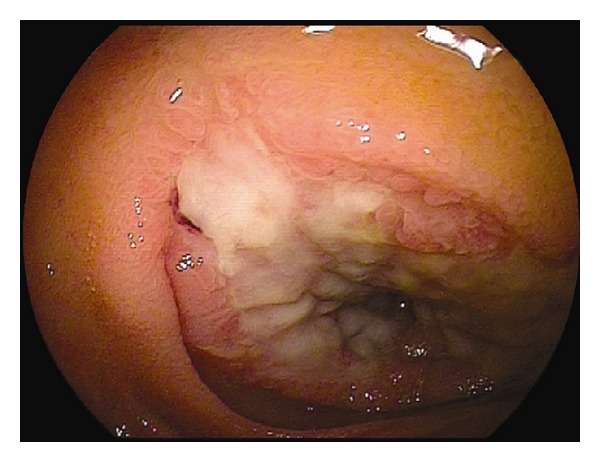
Double balloon enteroscopy. A large but shallow ulcer is seen in front of tight fibrous stricture of the distal jejunum.

**Figure 3:**
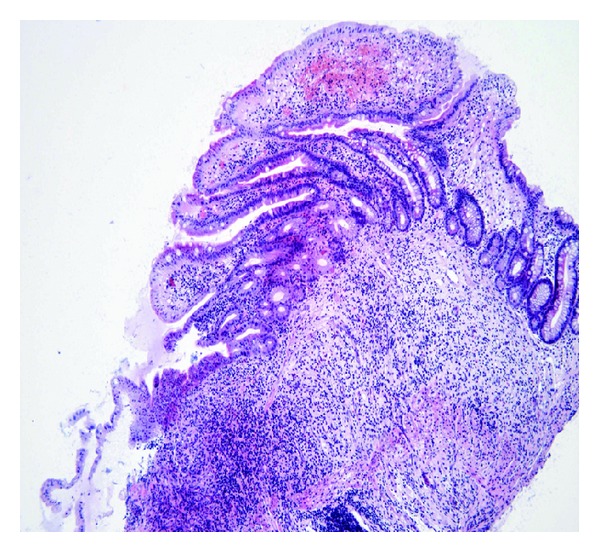
Increased content of collagen was found in interstitial tissue of the small bowel. Optical histology, hematoxylin-eosin staining. Original magnification 40x. Courtesy of Věra Tyčová, MD, the Fingerland Department of Pathology, Charles University, Faculty of Medicine and University Teaching Hospital, Hradec Králové.

**Figure 4:**
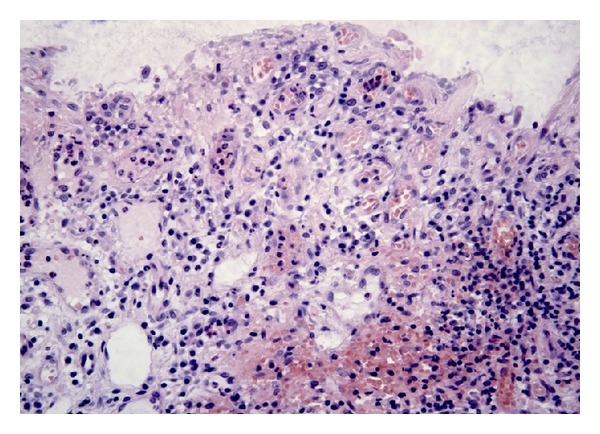
Small intestinal epithelium, impaired by extensive infiltration with plasmatic cells and lymphocytes. Optical histology, haematoxilin-eosin staining. Original magnification 100x. Courtesy of Věra Tyčová, MD, the Fingerland Department of Pathology, Charles University, Faculty of Medicine and University Teaching Hospital, Hradec Králové.

**Figure 5:**
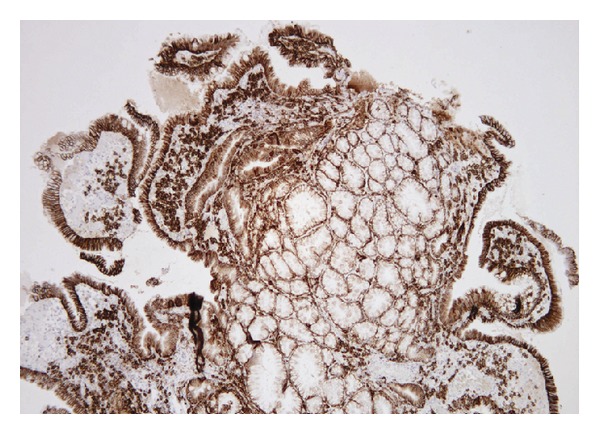
Prevailing infiltration with plasmatic cells was identified in all layers of the small intestine. Immunohistochemistry, anti-CD138 staining. Original magnification 100x. Courtesy of Věra Tyčová, MD, the Fingerland Department of Pathology, Charles University, Faculty of Medicine and University Teaching Hospital, Hradec Králové.

**Figure 6:**
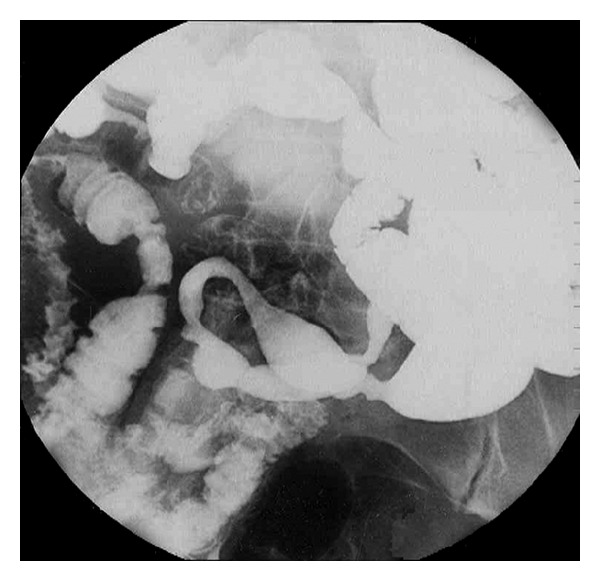
Enteroclysis. Multiple persisting stenoses of the small intestine caused by fibrous strictures. Courtesy of Zdenĕk Vacek, MD, Department of Radiology, Charles University Faculty of Medicine and University Teaching Hospital, Hradec Králové.

In CMUSE, there are small intestinal stenoses, shallow ulcers, and mixed inflammatory infiltrate at histology (plasma cells, monocytes, neutrophils, and eosinophils), see Figures 1, 2, 3, 4, 5, and 6. 

## 6. Diagnostics and Differential Diagnosis

Diagnosis of CMUSE is based on history, clinical features, CT/MR enteroclysis, small intestinal endoscopy, and histology of the small bowel (see Figures 1–6). 

The small intestinal ulcers in CMUSE/CNSU occur predominantly in the ileum; the terminal ileum is usually spared. The ulcers are usually multiple (more than 20). The small intestinal lesions never progress to cobble-stone appearance, fissure or fistula formation. The ulcers are restricted to the mucosa or submucosa; they never extend to the proper muscular layer. The mucosal lesions are characterised by infiltration of plasma cells, lymphocytes, and eosinophils [2].

There are no specific laboratory tests for the diagnosis of CMUSE. The faeces are positive for occult blood. Peripheral blood test reveals iron-deficiency microcytic anaemia. Some patients may manifest with hypoproteinemia and hypoalbuminemia. C-reactive protein and other acute inflammatory reactants are usually within their normal ranges or slightly increased [2].

Yao et al. proposed diagnostic criteria of CMUSE/CNSU in 2004 (see Table 1) [2]. 

Wireless capsule endoscopy was complicated by retention of the capsule in one of our cases of CMUSE [44] and was also reported by others [18]. Spontaneous disintegration of a retained video capsule was reported by our group previously [44]. 

In differential diagnosis of CMUSE, first of all, Crohn's disease must be excluded [2, 3, 18, 29, 45–47], see Table 2. Crohn's disease may occur in the jejunum without active disease elsewhere, but this seems to be uncommon, and jejunal involvement is usually associated with other clinical and pathological features of Crohn's disease [29]. CMUSE is characterized by mixed inflammatory infiltrate at histology (mostly plasma cells) [18, 44]. 

In further differential diagnosis of CMUSE, other small intestinal diseases must be excluded, too, especially NSAIDs-induced enteropathy [2, 3, 18, 46–50], tuberculosis and other chronic infections of the small bowel [18, 33, 51], Behçet disease [52–54], drug-induced small intestinal injury (thiazides, potassium chloride) [2], and malignancies [3]. It is a well-known fact, that the majority of extranodal malignant lymphoma involves the gastrointestinal tract. Not only polypoid and diffuse types of this disease, but also the ulcerative ones can be detected in the small bowel [55]. Usually either diffuse large B-cell lymphoma or mucosa-associated lymphoid tissue (MALT) lymphoma is confirmed by histology [56]. Multiple nonspecific ulcers in the small and large intestines occurred during tocilizumab therapy for rheumatoid arthritis [57]. 

 It is also obligatory to distinguish CMUSE from other nonfrequent pathological conditions. We are convinced, that CMUSE is distinct from chronic ulcerative jejunitis [58, 59], collagen sprue [60], and autoimmune or eosinophilic enteritis [18, 61]. Similarly, nonspecific small intestinal ulcers [18, 62–64] should not be considered to be CMUSE if multiple stenoses of the small bowel are absent [3]. 

## 7. Therapy 

No causal treatment is available so far. Therapy with systemic glucocorticosteroids is the treatment of choice [1–3]. Dose of corticosteroids differs interindividually; some patients need systemic glucocorticosteroids (20 mg prednisone per day) and some do profit from topic steroids (budesonide 9 mg per day) [3]. However, most patients develop corticosteroid dependence [1–3, 25]. Enteral or parenteral nutrition together with iron supplementation are transiently effective. Treatment with oral 5-aminosalicylic acid and azathioprine was ineffective. This therapy failed to induce mucosal healing or to prevent small intestinal strictures [2].

Multiple small intestinal fibrous strictures were previously resected surgically [1, 2, 24]; nowadays, they can be treated endoscopically by means of deep enteroscopy [3, 45]. Balloon dilatation of small intestinal non-ulcerated stenoses, if combined with corticosteroids, leads to disappearance of colicky abdominal pain and stenoses (verified on deep enteroscopy) [1, 45]. With the intention to avoid short bowel syndrome, surgery is the last choice of treatment in contemporary medicine with the availability of deep enteroscopy (when severe ileus episode occurs and cannot be managed by the pharmacology means of treatment, that is, intravenously administrated glucocorticosteroids) [25]. 

Recently, de Schepper et al. [65] reported that they had induced remission in CMUSE by anti-TNF-alpha therapy (using infliximab). 

## 8. Prognosis 

Prognosis of CMUSE remains uncertain. Patients were previously referred to surgery because of symptomatic small intestinal strictures. However, the postoperative recurrence rate was high [1]. Matsumoto et al. [2] reported a single case of CMUSE followed up for 40 years since 1963. This patient was operated seven times because of recurrence of tight stenoses and several resections of the small bowel had to be performed [2]. 

## 9. Conclusions

In conclusion, CMUSE, although a rare condition affecting the small bowel, should always be considered when chronic or relapsing subileus episodes result from multiple small intestinal strictures, and multiple shallow ulcers of the small bowel are found (in the absence of Crohn's disease, NSAID use, or chronic small intestinal infection). Deep enteroscopy enables precise diagnostic work; possible endoscopic treatment of stenoses may obviate the need for surgery and prevent excessive small bowel resections. 

## Figures and Tables

**Table 1:** Diagnostic criteria of CMUSE/CNSU.

(1) Persistent and occult blood loss from the GI tract except during bowel rest or postoperative period.	
(2) Confirmation of characteristic small intestinal lesions by macroscopy, radiography, or enteroscopy.	
(2.1) Circular or oblique in alignment.	
(2.2) Sharply demarcated from surrounding normal mucosa.	
(2.3) Geographic or linear in shape.	
(2.4) Multiplicity in number with <4 cm distance from each other.	
(2.5) Ulcers not reaching proper muscular layer.	
(2.6) Scarred ulcers presumed to be the healing stage of those characterised by (2.1)–(2.5)* in cases treated by bowel rest.	

Adopted from Matsumoto et al. [2].

*Depicted as symmetric and eccentric rigidity under small-bowel radiography, and concentric or non-concentric stricture under enteroscopy.

**Table 2:** Differentiation of CMUSE from Crohn's disease.

Absence of clinical or laboratory features of an inflammatory syndrome	
Absence of small intestinal transmural inflammatory process or ulceration	
Absence of small intestinal giant-cell granulomatous inflammatory process	
Absence of small intestinal fistula formation despite recurrent chronic disease	
Absence of disease in other parts of gastrointestinal tract (i.e., stomach or colon)	
Absence of most extraintestinal features of Crohn's disease (e.g., skin manifestations)	

According to Freeman [29].
